# Short-Term Forecasting of Monkeypox Cases Using a Novel Filtering and Combining Technique

**DOI:** 10.3390/diagnostics13111923

**Published:** 2023-05-31

**Authors:** Hasnain Iftikhar, Murad Khan, Mohammed Saad Khan, Mehak Khan

**Affiliations:** 1 Department of Mathematics, City University of Science and Information Technology, Peshawar 25000, Pakistan; 2 Department of Statistics, Quaid-i-Azam University, Islamabad 45320, Pakistan; 3 Department of Statistics, Abdul Wali Khan University Mardan, Mardan 23200, Pakistan; 4 Faculty of Computer Sciences and Engineering, Ghulam Ishaq Khan Institute of Engineering Sciences and Technology, Topi, Swabi 23640, Pakistan; 5 Department of Computer Science, AI Lab, Oslo Metropolitan University, P.O. Box 4 St. Olavs Plass, 0130 Oslo, Norway

**Keywords:** monkeypox virus, short-term forecasting, machine learning models, time series models, filtering and combining technique

## Abstract

In the modern world, new technologies such as artificial intelligence, machine learning, and big data are essential to support healthcare surveillance systems, especially for monitoring confirmed cases of monkeypox. The statistics of infected and uninfected people worldwide contribute to the growing number of publicly available datasets that can be used to predict early-stage confirmed cases of monkeypox through machine-learning models. Thus, this paper proposes a novel filtering and combination technique for accurate short-term forecasts of infected monkeypox cases. To this end, we first filter the original time series of the cumulative confirmed cases into two new subseries: the long-term trend series and residual series, using the two proposed and one benchmark filter. Then, we predict the filtered subseries using five standard machine learning models and all their possible combination models. Hence, we combine individual forecasting models directly to obtain a final forecast for newly infected cases one day ahead. Four mean errors and a statistical test are performed to verify the proposed methodology’s performance. The experimental results show the efficiency and accuracy of the proposed forecasting methodology. To prove the superiority of the proposed approach, four different time series and five different machine learning models were included as benchmarks. The results of this comparison confirmed the dominance of the proposed method. Finally, based on the best combination model, we achieved a forecast of fourteen days (two weeks). This can help to understand the spread and lead to an understanding of the risk, which can be utilized to prevent further spread and enable timely and effective treatment.

## 1. Introduction

In today’s world, humans have made incredible advances in the fields of science, technology, and artificial intelligence (AI) by inventing remote-controlled drones, automated responsive robots, and self-driving cars. However, they continue to face numerous natural disasters challenges, such as floods, earthquakes, and droughts, as well as novel viral diseases, including the coronavirus infectious disease 2019 (COVID-19) and the monkeypox virus (MV). The COVID-19 pandemic was one of the worst disasters in human history and caused 6.8 million deaths around the world as of 17 February 2023 [1]. Moreover, it is on the decline and is in the endemic phase, while the world is about to face another crisis in the form of a new viral disease outbreak of MV. MV is transmitted from one human to another through physical contact with the one who is infected, with contaminated matter, or with infected animals. The symptoms of MV include a rash on the skin, a mucosal lesion, muscle aches, headaches, back pain, fever, low energy, swollen lymph nodes, and a fever that can last for around 2 to 4 weeks. It can be difficult to identify and distinguish MV due to its similarity to other infectious diseases. It is important to differentiate MV from chickenpox, bacterial skin infections, capsules, herpes, scabies, other sexually transmitted infections, and medicine-related allergies. Therefore, diagnostic tests are necessary to distinguish it from other similar diseases, to obtain treatment as soon as possible, and to stop the spread. The most preferred laboratory diagnostic test for MV is the detection of viral DNA by polymerase chain reaction. MV is an infectious disease caused by the MV, a species of the Orthopoxvirus *genus* and a causative agent of smallpox. There are two different clades of MV: Clade I and Clade II. MV was first detected in Copenhagen, Denmark, in 1958 [2,3].

Meanwhile, the first formally documented case was observed in a nine-year-old child in the Democratic Republic of the Congo (DRC) in 1970 [4,5]. Initially, the MV outbreak was limited to the continent of Africa but gradually spread to North America and Europe. Furthermore, confirmed MV cases were recorded in African countries, including one case in Cote d’Ivoire, one in Cameroon, three cases in Nigeria, four in Liberia, and thirty-eight in Congo, between 1970 and 1979. In the year 1986, the total confirmed cases reached 400, with a 10% mortality rate. Moreover, between 1991 and 1999, some small outbreaks were also observed in West Africa and the equatorial central region, and Congo alone observed 500 cases. After that, the continent of Africa witnessed a decline and an endemic stage of the MV outbreak [6,7]. Since 2005, thousands of suspected MV infectious cases have been recorded every year in the DRC. In 2017, MV re-emerged and spread among the people of Nigeria through travelers across the country. However, in May 2022, the sudden increase and rapid spread of the MV outbreak were seen across Europe, America, and all six regions of the WHO, with the reporting of around 87 thousand confirmed cases and 112 deaths in 110 countries. This variant of MV was due to Clade I of MV, and it was detected in a refugee camp in the Republic of Sudan. Later, many countries on different continents, including Asia, Australia, Africa, South America, North America, and Europe, witnessed the same outbreak of MV.

The MV outbreak was declared a “public health emergency of international concern” (PHEIC) by the World Health Organization (WHO) after reporting 82,000 confirmed cases on 23 July 2022 [8]. Recently, the multi-country MV outbreak has attracted global attention, as a cumulative total of 86,017 confirmed cases and 97 deaths have been reported as of 17 February 2023, worldwide in over 111 countries [9,10]. On 11 May 2023, the WHO declared that the emergence of the MV outbreak of international concern in multi-countries is over [11]. The WHO also published a response plan and guidance documents on strategic preparedness to curb the spread of MV. The proper diagnostics, surveillance, risk management, and engagement of the community are most important to stop the spread and eliminate the human-to-human spread of MV. MV poses a major threat to global health and the economy due to the non-availability of a dedicated vaccine, cure, and on-time detection; therefore, it needs community awareness, the education of health workers, and timely forecasting to prevent its spread [12]. For this purpose, time series and machine learning models are needed for modeling and forecasting the daily cumulative deaths and confirmed cases of MV to assist governments and stakeholders in controlling the spread.

In the past, several statistical and machine learning (ML) models have been widely used by researchers for the prediction of various diseases and pandemics [13,14,15,16,17,18,19]. There has been tremendous progress in the use of AI tools to detect, diagnose, and classify diseases [20,21]. Hence, it is possible to implement AI models for the disease classification, monitoring, and forecasting of MV outbreaks [22,23]. The researchers in [24] comparatively evaluated the performance of machine-learning-based multilayer perception models (MLP) and (autoregressive integrated moving average) ARIMA models for the forecasting of the cumulative confirmed cases of MV and found that MLP with a sigmoid function outperformed the traditional ARIMA model in forecasting the actual cases of MV. Further, the human historical monkeypox cases were used for the prediction of the MV transmission rate by the application of stack ensemble learning (SEL) and ML techniques including random forest, adaptive boosting regression (Adaboost), and gradient boosting (Gboost). The experimental results revealed that the SEL outperformed the other used algorithms [25]. For seven-day forecasting of MV cases in the USA, the five ML and time series models, including ARIMA, long short-term memory (LSTM), prophet, neural prophet, and a stacking model, were used, and 95% accuracy was achieved in the neural prophet output [26]. The researchers in [27] proposed an optimized hybrid deep learning approach based on the Al-Biruni earth-radius-based (BER) optimization technique and an optimized LSTM for forecasting confirmed cases of MV outbreaks with high accuracy and low error. Furthermore, researchers have analyzed the spread of MV across multiple countries by using various ML models, including linear regression (LR), random forest (RF), elastic net regression (EN), convolutional neural network (CNN), and artificial neural network (ANN), and found the CNN outperformed the other methods used in forecasting MV outbreaks. They have also used time series models, ARIMA, and seasonal autoregressive integrated moving averages (SARIMA) to measure the events’ occurrence over time [28]. In further studies, the ARIMA and feedforward neural network (FFNN) were used for the daily confirmed and death cases of MV outbreaks for the next two months; their forecasted estimates were around 87,276 confirmed and 94 deaths up to 31 January 2023, with a 95% confidence interval [29]. The authors in [30] performed the sentiment analysis of tweets related to the illness of MV by analyzing the three possible responses of positive, negative, and neutral with 94% accuracy via the proposed hybrid deep learning approach based on CNN-LSTM. The researchers in [31] conducted a study for a short-term forecast for 10 weeks of calibration by applying n-sub-epidemic models and found an accurate prediction of the declining trend in MPV cases globally and country-specific cases in the last subsequent period of ten days. Their findings also revealed that the population with behavioral modifications and increased immunity were at higher risk of being affected by viruses. Further, the authors in [32] implemented a lag-correlation analysis, a gradient causality test, and a vector autoregression model to forecast the monkeypox epidemic in 20 countries and found a robust association between 13 days of priority and daily confirmed cases globally.

In this paper, we propose a novel filtering and combination technique for the accurate and efficient short-term forecasting of cumulative confirmed cases of MV. The proposed methodology is based on various filters and machine learning models. The steps of the proposed methodology are as follows: First, we decompose the original time series of the cumulative confirmed cases into new subseries, that is, the long-term trend series and residual series, using the two proposed and one benchmark filter, including the regression spline filter (RSF), smoothing spline filter (SSF), and the Hodrick–Prescott filter (HPF). Then, to predict the decomposed subseries, we consider five well-known machine learning models, including artificial neural network (ANN), support vector machine with two kernels (linear (SVM1) and spline (SVM2)), random forest (RF), decision tree learning (DT), and all their possible combination models. Hence, we combine the individual predictive models directly to obtain the final predictions one day ahead of the cumulatively infected cases of MV. The contributions of this work can be summarized as follows: A novel filtering and combination approach is proposed based on different filters and various combinations of ML models to improve the accuracy of the MV-confirmed-case day-ahead forecasts. First, we verify the performance of the proposed filters compared to the standard benchmark filter method using accuracy measures and a statistical test. Second, within the proposed methodology, we compare the performance of the different combinations of the considered machine learning models using two proposed filters and a benchmark filter. Third, the proposed final combination model is compared with the standard time series and the ML models, and the comparative results are noted. The noted results showed that the proposed final model is highly accurate and efficient for forecasting the daily confirmed cases of MV as compared to the benchmark models. Finally, the proposed methodology can be generalized and tested for other datasets.

The rest of the paper is organized as follows: Section 2 describes the general procedure of the proposed filtering and combination forecasting methodology. Section 3 provides an empirical application of the proposed modeling framework using the daily cumulative time series of the MV. Section 4 comprises a discussion of the proposed best combination model versus some of the best time series and machine learning models. Finally, Section 5 addresses the concluding remarks and future research directions.

## 2. The Proposed Filtering and Combination Technique

This section explains the proposed filtering and combination forecasting technique for the short-term cumulative confirmed cases forecast. To achieve this, the time series of the cumulative confirmed cases ($N_{t}$) is decomposed into two subseries: the long-term nonlinear trend ($L_{t}$) and a residual subseries ($R_{t}$) using two new proposed filters and a considered benchmark filter, including the regression spline filter, the smoothing spline filter, and the Hodrick–Prescott Filter. The mathematical representation of the decomposed subsequence is formulated as

(1) $$N_{t}=L_{t}+R_{t}.$$

Therefore, for the modeling and forecasting purposes, the long-run nonlinear trend $L_{t}$ is a function of time t and the residual subseries, which describes the short-run dependence of the cumulative series and is obtained by $R_{t}=\left(N_{t}-L_{t}\right)$. Therefore, the new proposed filters and the benchmark filter are described in the following subsection.

- Regression Spline Filter

A regression spline is a general nonparametric approximation of $N_{t}$ by a piecewise m^th^ degree polynomial, estimating a subinterval bounded by a series of m points (called knots). Any spline function U(N) of order q can be defined as a linear combination of functions $U_{i}\left(N\right)$ called basis functions, whose formula is given by increase.

(2) $$U\left(N\right)=\sum_{i=1}^{t+m+1}\alpha_{i}U_{i}\left(N\right).$$

The unknown parameter is $\alpha_{i}$, estimated by the ordinary least squares method. The most important choices are the number of nodes and their positions that define the smoothness of the approximation. In this work, we used cross validation to estimate these quantities.

- Smoothing Splines Filter

To meet the requirements for resolving the knot regions, spline features can be predicted using a least-squares penalty environment to limit the sum of squares. Hence, the equation can be written as

(3) $$\sum_{j=1}^{N}{\left(N_{t}-U\left(N\right)\right)}^{2}+\lambda \int {\left(U^{\prime \prime }\left(N\right)\right)}^{2}\mathrm{dt},$$

where $\left(U^{\prime \prime }\left(N\right)\right)$ is the second derivative of U(N). The first term describes the goodness of fit, and the second term penalizes the coarseness of the function by the smoothing parameter λ. Moreover, the selection of smoothing parameters is a difficult task and is performed by cross-validation methods in this work.

- Hodrick–Prescott Filter

To assess the performance of the two proposed filters, they are compared to a standard benchmark filter, the Hodrick–Prescott Filter (HPF). The HPF is used to obtain a smoothed-curve representation of a time series that is more complex for long-term than short-term fluctuations. The adjustment of the sensitivity of the trend to short-term fluctuations is achieved by modifying a multiplier λ. Let $N_{t}$ (t = 1,2,...., N) be denoted the time series data. The series $N_{t}$ is made up of a trend component denoted by $\tau_{t}$, and an error component, denoted by ε; therefore, the equation is

(4) $$N_{t}=\tau_{t}+\varepsilon_{t}.$$

Here, we insert this into in a long-term trend component that can be estimated by minimizing the following expression,

(5) $$min_{t}\left(\sum_{t=1}^{M}{\left(N_{t}-\tau_{t}\right)}^{2}+\lambda \sum_{t=2}^{M-1}{\left[\left(\tau_{t+1}-\tau_{t}\right)-\left(\tau_{t}-\tau_{t-1}\right)\right]}^{2}\right).$$

In the above equation, the first term is the loss function, and the second term is a plenty term λ multiplied by the sum of the square of the trend component second difference, which penalizes the variation in the growth rate of the trend component.

**Figure 1:**
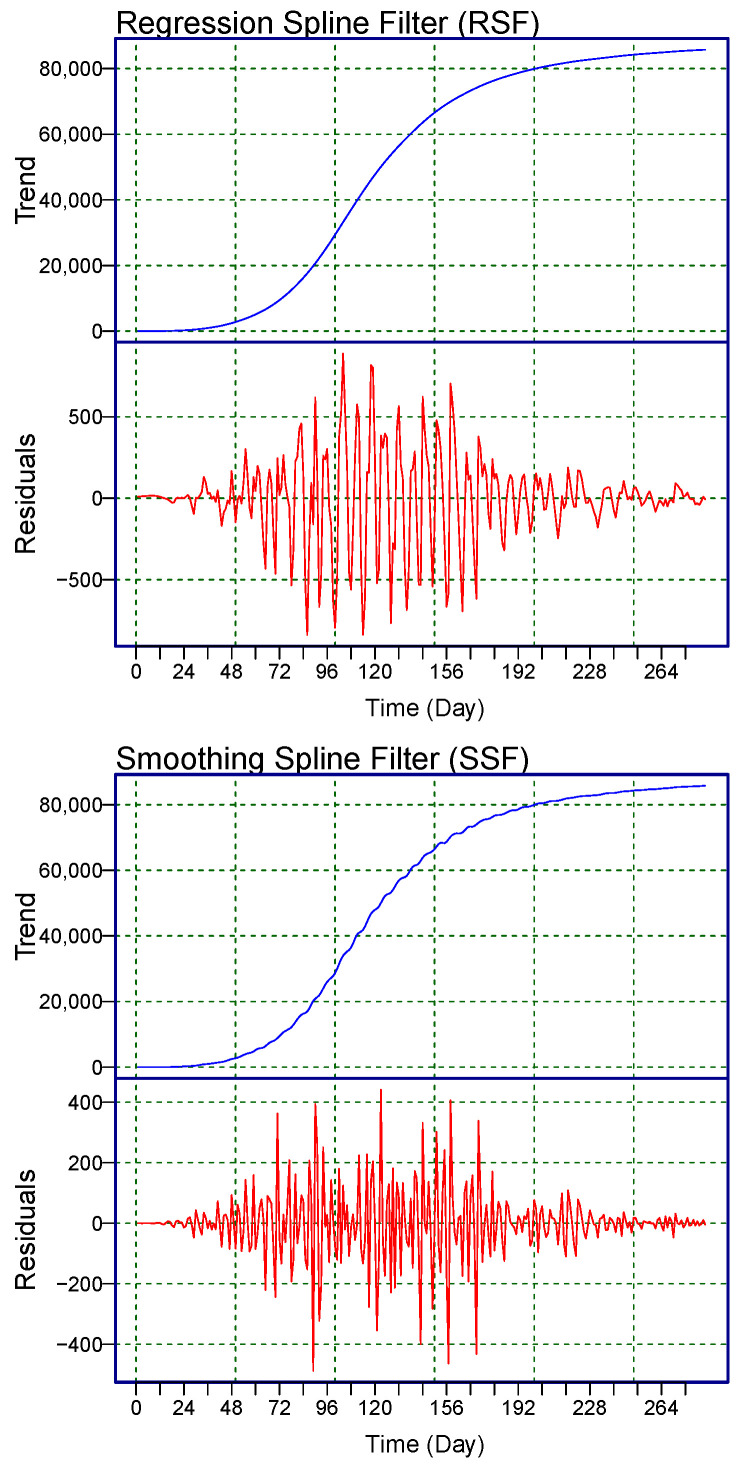

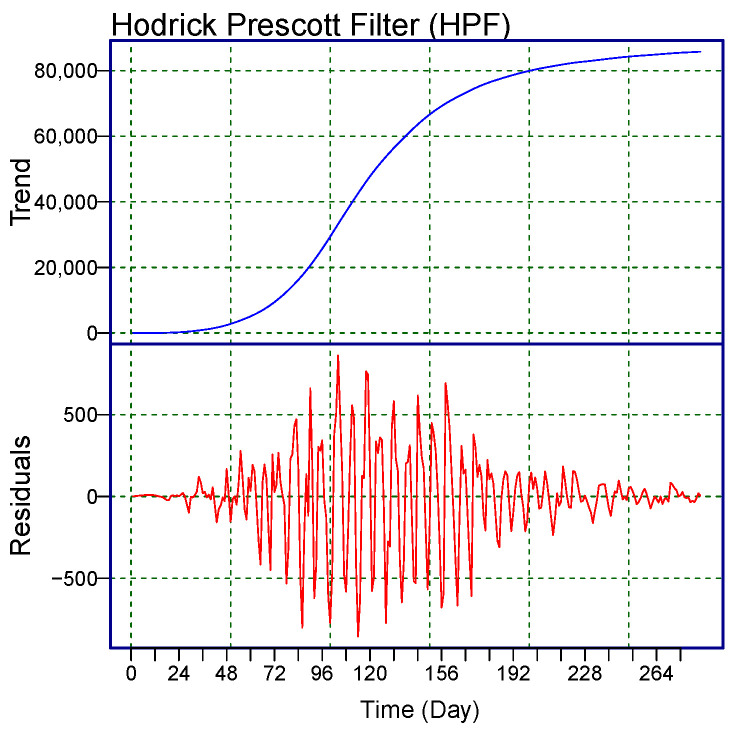
World Monkeypox Virus Data: the daily confirmed cases of the monkeypox virus are filtered by the two proposed filters: (**top**) RSF, (**middle**) SSF, and the benchmark filter HPF (**bottom**). Within each subfigure, the top panel shows the long-term trend (blue curve-$L_{t}$), and the bottom panel shows the residual part (red curve-$R_{t}$).

To graphically demonstrate the performance of the proposed filters and the considered benchmark filter (HPF) presented above, the new subseries are shown in Figure 1. In each subfigure, at the first position is a nonlinear increasing trend ($L_{t}$) subseries, and in the second position is a residual ($R_{t}$) subseries. From the figure, we observe that the proposed and the benchmark filters decomposed ($N_{t}$) and captured the long-term nonlinear trend well.

### 2.1. Modeling to Filtered Series

Once the filtered subseries are extracted from the time series of the daily cumulative confirmed cases using the two new proposed filters and a benchmark filter, we estimate the extracted subseries using five standard machine learning models, including artificial neural network, support vector machine with two kernels (linear and spline), random forest, and decision tree learning. Therefore, all the considered models are explained as follows:

- Nonlinear autoregressive neural network

A nonlinear autoregressive neural network (ANN) is a type of machine learning model that is useful for forecasting the future values of an input variable. The ANN network predicts the future values of a time series based on its history using a re-feeding mechanism, in which an expected value can be used as an input for new predictions at later points in time [33]. The network is built and trained in an open loop, using actual target values as feedback to ensure greater training accuracy. The network is converted into a closed loop after training, and the predicted values are used to supply new feedback inputs to the network.

Mathematically, the model predicts the future values of a time series $N_{t}$ based on its historical values $N_{t-1},N_{t-2},\ldots ,N_{t-d}$, where d is the time delay parameter. The backpropagation algorithm is used to train the network, and the steepest descent method is used to minimize the square error between the actual and predicted values.

- Support Vector Machine

The statistical learning theory and the concept of structural risk reduction, which were first introduced by Cortes and Vapnik in 1995, serve as the foundation for the machine learning algorithm known as support vector machine (SVM). One of the most widely used methods for supervised learning, the SVM is used to solve classification and regression issues [34]. The SVM is quick, easy to operate, consistent, and generates accurate results. On the other hand, SVM models employ a variety of fundamental kernel operations. The functions can be categorized as a polynomial, linear, sigmoid, and exponential radial basis function and a radial basis function. Complex nonlinear decision boundaries can be modeled by the SVM technique. In addition, the SVM model is effective in time series forecasting because it can resolve issues with nonlinear regression estimates. The formula listed below determines the SVM model for a given dataset.

Consider an n set of data (x1, y1), …, (xn, yn), where xi is the ith input vector, and yi is the corresponding desired output. Because i = 1, 2, …, n, where n is the size of the sample, the estimating function assumes the following form:

(6) $$\delta_{n}=\varpi .\phi \left(\delta_{n}\right)+\mu ,$$

where ϖ is the weight vector, μ is the bias, $\phi (\delta_{n})$ is the high-dimensional feature space nonlinearly mapped from the input space, and (·) represents the inner product. This work considers two kernels, the linear (SVM1) and spline (SVM2) kernels.

- Random Forest Model

A well-known machine learning algorithm that belongs to the supervised learning class is called the random forest (RF). Random forest often referred to as random decision forest, is an ensemble learning technique for classification and regression that operates by putting several decision trees through training. The RF algorithm determines the outcome based on the decision trees’ predictions [35]. The output of different trees is averaged to produce forecasts. As the number of trees increases, the accuracy of the result increases. The more trees in the forest, the more accurate it is, and the issue of overfitting is avoided. To train tree learners, the random forest training algorithm employs the widely used method of bootstrap aggregation or bagging [36]. Bagging repeatedly (M times) takes a random sample with replacement of the training set and fits trees to these samples given a training set $Y=y_{1},\ldots ,y_{n}$ with responses $X=x_{1},\ldots ,x_{n}$.

For m=1,…,M:

1. m training instances from Y, X are sampled with replacement; they are referred to as $Y_{m},X_{m}$.

2. On $Y_{m},X_{m}$, we train a classification or regression tree $f_{m}$. After training, summing the predictions from all the various regression trees on $y^{\prime }$ can be used to make predictions for the unseen samples $y^{\prime }$:

(7) $$\hat{f}=\frac{1}{M}\sum_{m=1}^{M}f_{m}\left(y^{\prime }\right).$$

This bootstrap technique enhances the model performance by reducing the model variance without increasing the bias. The majority vote is used in classification tree cases.

- Decision Tree Learning

Data mining frequently uses the decision tree learning technique [37]. The goal is to develop a model that predicts the value of a target parameter given a set of input parameters. A tree can be trained to learn by subdividing the source dataset depending on an attribute value test [38]. With the use of a recursive partitioning process, a decision tree algorithm divides a training dataset, $Y=\{Y_{1},Y_{2},\ldots ,Y_{N}$} → $X=\{X_{1},X_{2},\ldots ,X_{N}$}, to create a model. Note that each observation $Y_{i}$→$X_{i}$ could include $M_{f}$ features, $Y_{i}=\left(Y_{i1},Y_{i2},\ldots ,Y_{iM_{f}}\right)$. If the data at node m are Q with $M_{m}$ observations, then for each candidate partition $\pi =(f,t_{m})$ consisting of a feature f and threshold $t_{m}$, the data Q are split into $Q_{left}\left(\pi \right)$ and $Q_{right}\left(\pi \right)$ subsets, such that

$$Q_{left}\left(\pi \right)=\left(Y,X\right)|Y_{f}<t_{m},$$

and

$$Q_{right}\left(\pi \right)=Q\setminus Q_{left}\left(\pi \right).$$

The impurity at node n after each partition is defined as:

$$I\left(Q,\pi \right)=\frac{M_{left}}{M_{m}}G\left(Q_{left}\left(\pi \right)\right)+\frac{M_{right}}{M_{m}}G\left(Q_{right}\left(\pi \right)\right).$$

G (…) is the impurity function in this situation.

$$G\left(Q\right)=\frac{1}{M_{m}}\sum_{i\in M_{n}}{\left(X_{i}-{\bar{X}}_{m}\right)}^{2},$$

$${\bar{X}}_{n}=\frac{1}{M_{m}}\sum_{i\in M_{n}}X_{i}.$$

By reducing the impurity I(Q,π), the decision tree model’s parameters are chosen.

$$\pi^{*}=argmin_{\pi }I(Q,\pi ).$$

Recursively, the identical partitioning operation is carried out for the subsets $Q_{left}\left(\pi^{*}\right)$ and $Q_{right}\left(\pi^{*}\right)$ up until the maximum permitted depth is reached, or there is only one observation left ($M_{m}$ = 1). The model may then be used to forecast the value of a target variable ($X_{test}$) based on the independent variables ($Y_{test}$) after the decision tree has been trained with the training dataset, Y and X.

In the current study, we denote each combined model with each filter method by RSF${}_{R_{t}}^{L_{t}}$, where the $L_{t}$ at the top right is associated with a nonlinear trend subseries, and the $R_{t}$ at bottom right is associated to the residual subseries. In the forecasting models, we assign a code to each model: “0” for the ANN, “1” for the SVM1, “2” for the SVM2, “3” for the RF, and “4” for the DT. For example, RSF${}_{1}^{0}$ represents the estimate of the long-term trend ($L_{t}$) with the ANN, and the residual series ($R_{t}$) estimated using the SVM1. The individual forecast models are summed to obtain the final one-day-ahead cumulative confirmed cases to forecast.

(8) $$\begin{array}{ccc} {\hat{N}}_{t+1} & = & \left({\hat{L}}_{t+1}+{\hat{R}}_{t+1}\right). \end{array}$$

### 2.2. Accuracy Measures

In the literature, many researchers have used various accuracy measures and statistical tests to check the performance of predictive models [39]. However, in this work, we initially use four accuracy mean errors for the proposed evaluation of all sixteen combination models, such as the root mean square percentage error (RMSPE), the root mean square error (RMSE), the mean absolute error (MAE), and the mean absolute error percentage (MAPE). The mathematical equations for these mean errors are the following:

(9) $$\mathrm{RMSPE}=\sqrt{\frac{1}{T}\sum_{t=1}^{T}{\left(\frac{|N_{t}-{\hat{N}}_{t}|}{|N_{t}|}\right)}^{2}}\times 100,$$

(10) $$\mathrm{MAPE}=\frac{1}{T}\sum_{t=1}^{T}\left(\frac{|N_{t}-{\hat{N}}_{t}|}{|N_{t}|}\right)\times 100,$$

(11) $$\mathrm{MAE}=\frac{1}{T}\sum_{t=1}^{T}\left(|N_{t}-{\hat{N}}_{t}|\right),$$

(12) $$\mathrm{RMSE}=\sqrt{\frac{1}{T}\sum_{t=1}^{T}{\left(N_{t}-{\hat{N}}_{t}\right)}^{2}},$$

where N is the number of observations in the dataset, ($N_{t}$) and (${\hat{N}}_{t}$) are the T${}^{\mathrm{th}}$ estimated and observed data points, respectively.

**Figure 2:**
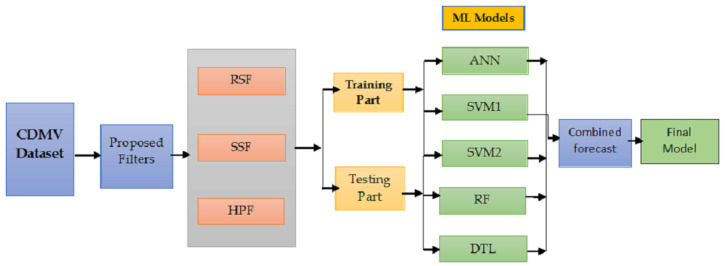
A flowchart of the proposed filtering and combination technique.

The layout of the proposed filtering and combination technique is shown in Figure 2.

## 3. Data Description and Case Study Results

**Figure 3:**
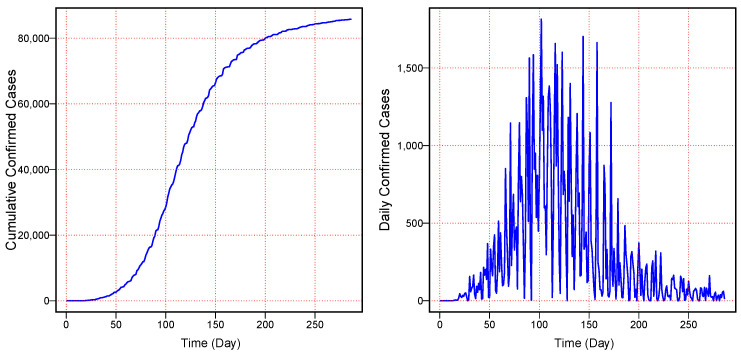
World Monkeypox Virus Data: the cumulative confirmed cases of monkeypox virus (**left**) and the daily confirmed cases of monkeypox virus (**right**) from 7 May 2022 to 10 February 2023.

The main aim of this work is to provide a short-term forecast of the cumulatively infected cases of MV using a dataset from the entire world. The dataset (daily cumulative confirmed cases) of MV was taken from the official website of “Our World in Data” from 7 May 2022 to 10 February 2023. The graphical presentation and the descriptive statistics of daily and cumulative confirmed cases can be seen in Figure 3 and Table 1. Figure 3 (left) shows the daily cumulative infected cases of MV, and Figure 3 (right) shows the daily new infected cases of MV. Further, Figure 3 (left) shows an increasing nonlinear curve, while Figure 3 (right) shows low infected cases at the start and end of the considered series and high cases in the middle of the considered series. It was confirmed that at the start, MV had a low number of infections, and with time, it gradually increased, but after that, it again showed a gradual decline in the infected cases. On the other hand, the descriptive statistics are tabulated in Table 1. This table shows that the minimum number of confirmed cases of MV was zero, and the maximum number was 1814. Moreover, the average number of infected cases was 300 throughout the world, while the variation among the infected cases was observed by the standard deviation with a value of 406. Therefore, the complete dataset of the daily cumulative confirmed cases covering 286 days was split; 7 May to 11 November 2022 (214 days) was used for model training, and 12 November 2022 to 10 February 2023 (72 days) was used for the one-day-ahead cumulative confirmed cases post-sample (testing) forecasts.

To obtain the forecast for the daily confirmed MV cases a day ahead, using the filtering and combination forecasting technique described in Section 2, the following steps were followed: first, the new two proposed filters and a benchmark filter were used to obtain a long-term nonlinear trend ($L_{t}$), and residual ($R_{t}$) time subseries. Second, the previously described five well-known machine learning models were applied to each subseries. Thereby, the models were estimated, and a day-ahead forecast for 72 days was obtained using the rolling window method. The final daily confirmed MV cases day-ahead forecasts were obtained using Equation (8). The accuracy measures RMSPE, RMSE, MPAE, and MAE were then used to evaluate and compare the performance of the models.

The actual time series of the daily cumulative confirmed MV cases ($N_{t}$) was divided into a long-term nonlinear trend ($L_{t}$) and a residual subseries ($R_{t}$), and two proposed filters and a benchmark filter were used in this work. Forecasts for these subseries were obtained using five machine learning models. To this end, combining the model and subseries forecasts, there were ($5^{L_{t}}\times 5^{R_{t}}$ = 25) different combinations for each proposed filter. Thus, there were two proposed filters (RSF and SSF) and one benchmark filter (HPF), for a total of 75 (3×25) models. For these 75 models, the out-of-sample forecast accuracy measures for one day ahead (RMSPE, RMSE, MPAE, and MAE) are tabulated in Table 2, Table 3 and Table 4. The results of the performance measures showed that the RSF${}_{1}^{2}$ model produced a better prediction than all the other models using the RS filter. The best forecasting model was RSF${}_{1}^{2}$, which produced 0.1452, 1.2083, 1010.7360, and 1207.9950 for the RMSPE, MAPE, MAE, and RMSE, respectively. However, the RSF${}_{1}^{1}$ and RSF${}_{1}^{3}$ models produced the second and third-best results. On the other hand, using the SS filter, the lowest forecast errors were found by the SSF${}_{1}^{2}$ model with the values of 0.1415, 1.2162, 1020.2140, and 1182.9890 for the RMSPE, MAPE, MAE, and RMSE, respectively. Notwithstanding, the second- and third-best results were achieved by the SSF${}_{1}^{1}$, and SSF${}_{1}^{0}$ models, respectively. In contrast, the benchmark filter (HPF) was outperformed by the proposed filters. Therefore, it was confirmed from Table 2, Table 3 and Table 4, within the proposed filtered and benchmark filters, the RS filter produced the lowest mean errors. Moreover, within all the possible combination models ($5^{L_{t}}\times 5^{R_{t}}$ = 25 = 75), the RSF${}_{1}^{2}$ was declared the best model.

**Figure 4:**
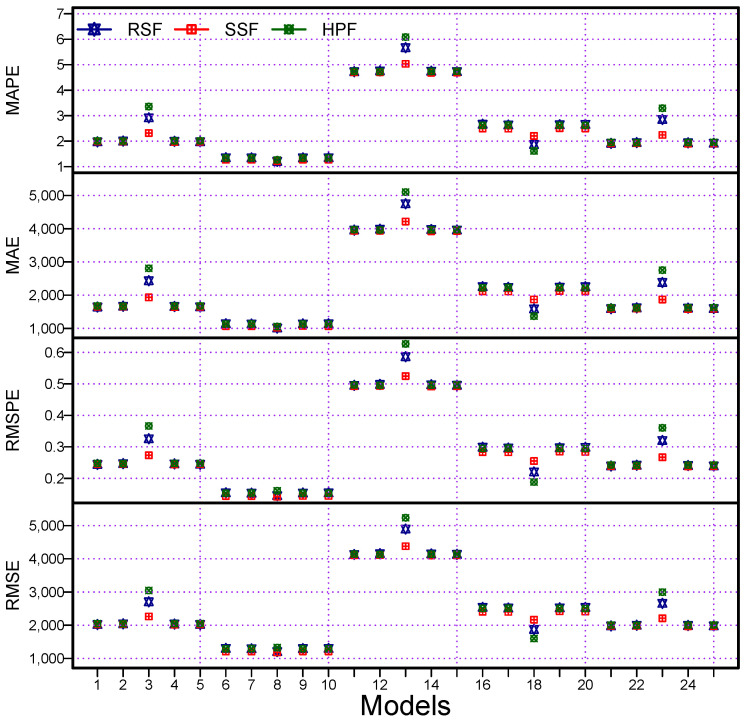
World Monkeypox Virus Data: accuracy measurement plots: MAPE (1st), MAE (2nd), RMAPE (3rd), and RMSE (4th), for all combination models using two proposed filters and a benchmark filter.

On the other hand, from the proposed filters (RSF and SSF) and the benchmark filter (HPF), one best combination model from each filter was selected and compared. The mean of the accuracy measures numerically are listed in Table 5 and graphically presented in Figure 4. From both presentations, it was confirmed that the RSF${}_{1}^{2}$ produced the lowest values (RMSPE = 0.1452, MAPE = 1.2083, MAE = 1010.7360, and RMSE = 1207.9950) among the best combination models. Finally, it was concluded that the proposed filter (RSF) resulted in more accurate forecasts than the other proposed filter and a benchmark filter. On the other hand, the RSF${}_{1}^{2}$ was confirmed as the best model.

Once the accuracy measures were calculated, the next step was to evaluate the dominance of these results. For this purpose, many researchers in the literature have performed the Diebold and Mariano test (DM) [40,41,42,43]. In this work, to verify the superiority of the proposed filtering and combination forecasting system results (accuracy measurements) presented in Table 2, Table 3 and Table 4, we performed tests by Diebold and Mariano (DM) on each pair of models [44]. The DM test results (*p*-values) are shown in Table 6. In contrast to the alternative that each entry in the table is p and that the column/row predictor accuracies are more accurate than the column/row predictor values of the hypothesis system, the null hypothesis was that there was no predictor. This table shows that among all the combination models, in Table 3, Table 4 and Table 5, the RSF${}_{1}^{2}$, SSF${}_{1}^{2}$, and SSF${}_{1}^{2}$ models were statistically superior to the others at the 5% significance level. In the same way, from the proposed filters (RSF and SSF) and the benchmark filter (HPF), one best model from each combination of filters was selected, and their accuracy error is listed in Table 5. Moreover, to verify the superiority of the best-selected models, the DM test results (*p*-values) are shown in Table 6; it was confirmed that the RSF${}_{1}^{2}$ was statistically superior to the others at the 5% significance level.

**Figure 5:**
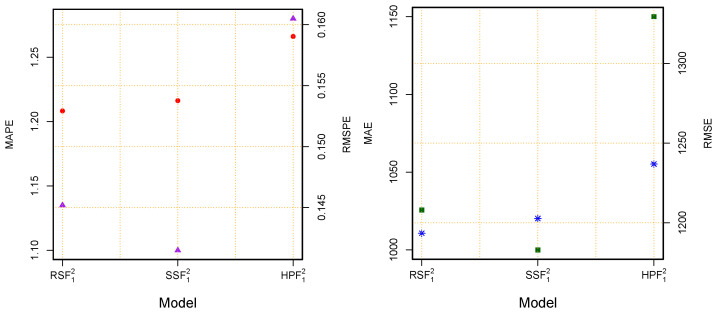
World Monkeypox Virus Data: accuracy measurement plots for the best three models, (**left**) (MAPE-red circle and RMSPE-purple triangle) and (**right**) (MAE-blue star and RMSE-green square) for the best three final models using the two proposed filtering methods and a benchmark filter.

Finally, the graphical representations of the performance measures for all 75 models are also shown in Figure 4, for the MAPE (1st), MAE (2nd), RMSPE (3rd), and RMSE (4th). In these plots, we can see that the proposed filters produced the highest accuracy (MAPE, MAE, RMSPE, and RMSE) when compared with the considered benchmark filter (HPF). However, within the proposed filters, the RSF obtained the highest accuracy. Moreover, within all the possible combination models (75), the RSF${}_{1}^{2}$ was declared as the best model. Therefore, from the descriptive statistics, statistical tests, and graphical results, we can conclude that the proposed forecasting methodology is highly accurate and efficient for the daily cumulative confirmed cases of MV forecasting. Additionally, the proposed filters had high accuracy and resulted in efficient forecasts when compared with the considered benchmark filter. Within the set of proposed filters, the RS filter produced a more precise forecast when compared with the alternatives. Moreover, it was confirmed from these visualizations that the RSF${}_{1}^{2}$ was statistically superior to the others (Figure 5).

## 4. Discussion

According to the results (the descriptive statistics, statistical test, and graphical analysis), the conclusion is that the final best combination model was the RSF${}_{1}^{2}$, which was highly accurate and efficient for the daily cumulative confirmed cases of MV. Hence, it is important to note that reported mean accuracy errors (RMSPE, MAPE, MAE, and RMSE) in this work were, for the best combination model (RSF${}_{1}^{2}$), relatively lower than the considered times series and machine learning benchmark models. The considered benchmark models were the following: three standard time series models: the autoregressive (AR), nonparametric autoregressive (NPAR), and autoregressive moving integrated average (ARIMA) models, and four popular machine learning models: ANN, SVM, DT, and RF. An empirical comparison of the proposed work’s best model with the other considered benchmarks (AR, ARIMA, NPAR, ANN, SVM, DT, and RF) models are presented numerically in Table 7. As we can see from Table 7 , the proposed best combination model (RSF${}_{1}^{2}$) in this work obtained significantly lower accuracy mean errors as compared to the time series and machine learning models. Additionally, to confirm the superiority of the proposed best combination model mentioned in Table 8, we performed a statistical test using the DM on each pair of models. The results (*p*-values) of the DM test are reported in Table 8, showing that the proposed models among all the considered time series and machine learning models were outperformed by our best model at the 5% significance level. To conclude, based on all of these results, the accuracy of the proposed forecasting methodology is comparatively high and efficient when compared with all the considered competitors.

Once the best models were assessed through descriptive statistics, statistical tests, and graphical analysis, we proceeded to future forecasting with the superior model. We used the RSF${}_{1}^{2}$ for the confirmed cases of MV, and forecast from 15 February to 26 February 2023 (two weeks) for the cumulative confirmed cases of MV. The forecasted values of the cumulative and daily confirmed cases from the MV are tabulated in Table 9. This table reveals that the daily confirmed cases increased in the first two days but decreased in the following three consecutive days, while on the rest of the days, there was an average increase of 45 per day. Furthermore, we predicted that during the next fourteen days (15 February to 26 February 2023), a total of 642 new cases of MV would be added globally. However, these forecasts continued to support an overall declining trend in the number of newly infected cases of MV around the world. Finally, to justify the superiority of the proposed final best model forecasting performance, we compared the cumulative infected cases of MV with the forecasted case by the proposed best model. To achieve this, we computed the percentage forecast error (PFE); the PEF is defined as PEF = (|forecasted value − actual value|/|actual value|) × 100. The values of the PFE are listed in the last column of Table 9. From this column, one can see that the forecasted values were relatively close to the actual values in terms of a low PFE. Therefore, our results offer valuable information to policymakers to guide the continued allocation of resources and inform mitigation efforts. Moreover, the forecasting exercise will help to understand the spread and lead to an understanding of the risk, which may be used to prevent further spread and enable timely and effective treatment.

On the other hand, actual-time forecasting in epidemic emergencies provides actionable information that governments can use to anticipate medical needs and strategize the intensity and composition of public health interventions. This study provides and evaluates accurate real-time short-term forecasts of monkeypox cases worldwide, with much higher case numbers reported in most epidemics. Our model continues to predict a slowdown in MV incidence globally. Overall, our model has proven useful in making short-term forecasts and capturing slowdowns and peaks in growth with reasonable accuracy.

## 5. Conclusions

The main aim of this work was to forecast the short-term transmission rate of the monkeypox virus. For this purpose, we proposed a novel filtering and combination forecasting approach to accurately forecast the daily cumulative confirmed monkeypox virus cases using the publicly updated monkeypox virus dataset. To this end, we first filtered the original time series of the cumulative confirmed cases into new subseries, that is, the long-term trend series and residual series, using two proposed and one benchmark filter. Then, to predict the decomposed subseries, we considered five well-known machine learning models and all their possible combinations. Hence, we combined the individual predictive models directly to obtain the final predictions for newly confirmed cases one day ahead. To verify the performance of the proposed filtering and combination methodology, four mean errors—two absolute errors, two relative errors, and a statistical test—were considered. The experimental results showed the efficiency and accuracy of the proposed filtering and combination forecasting system. Overall, this work was compared within the proposed forecasting technique with three filters: two proposed and a benchmark filter. Among these three filtering methods, the regression smoothing spline filter outperformed the rest. On the other hand, within all the possible combination models, the (RSF${}_{1}^{2}$), (SSF${}_{1}^{2}$), and (HPF${}_{1}^{2}$) were the first-, second-, and third-best models. In addition, the proposed final combination model (RSF${}_{1}^{2}$) had superior results (accuracy mean errors, graphical analysis, and statistical test) when all the time series and machine learning models were considered. Finally, based on the best-selected model (RSF${}_{1}^{2}$), we provided a forecast of the next fourteen days (15 February to 26 February 2023), which will help to understand the spread and lead to understanding the risk, which may be used to prevent further spread and enable timely and effective treatment.

As this study used only cumulatively confirmed monkeypox data, it could be extended to other variables (such as daily new infected cases and daily and cumulative death counts) to evaluate the performance of the proposed forecasting technique. Further, it could be used for the short-term forecasting of daily and cumulative COVID-19 confirmed cases, death counts, and recovered cases. On the other hand, the proposed forecasting techniques used only machine learning models; in the future, they will be extended by time series models, such as parametric autoregressive, nonparametric autoregressive, autoregressive integrated moving average models, etc.

## Figures and Tables

**Table 1:** Descriptive statistics for the daily confirmed cases of the monkeypox virus dataset.

S.No	Statistic	Value
1	Minimum	0
2	First Quartile	27.00
3	Median	125.50
4	Mean	299.90
5	Variance	164,481.70
6	Standard Deviation	405.56
7	Skewness	1.76
8	Kurtosis	2.39
9	Third Quartile	381.00
10	Maximum	1814.00

**Table 2:** World Monkeypox Virus Data: out-of-sample one-day-ahead mean forecast error for all combination models using the RS filter.

S.No	Models	RMSPE	MAPE	MAE	RMSE
1	**RSF${}_{0}^{0}$**	0.2442	1.9733	1642.6080	2022.8460
2	**RSF${}_{0}^{1}$**	0.2467	2.0019	1666.6090	2043.4370
3	**RSF${}_{0}^{2}$**	0.3258	2.9147	2434.1050	2706.2880
4	**RSF${}_{0}^{3}$**	0.2459	1.9922	1658.4570	2036.4830
5	**RSF${}_{0}^{4}$**	0.2445	1.9769	1645.6030	2025.4190
6	**RSF${}_{1}^{0}$**	0.1540	1.3494	1139.0140	1303.5960
7	**RSF${}_{1}^{1}$**	0.1529	1.3401	1130.9040	1293.1590
8	**RSF${}_{1}^{2}$**	0.1452	1.2083	1010.7360	1207.9950
9	**RSF${}_{1}^{3}$**	0.1532	1.3428	1133.2750	1296.6320
10	**RSF${}_{1}^{4}$**	0.1539	1.3481	1137.8680	1302.2670
11	**RSF${}_{2}^{0}$**	0.4953	4.7261	3957.2110	4130.2120
12	**RSF${}_{2}^{1}$**	0.4981	4.7562	3982.5270	4154.4740
13	**RSF${}_{2}^{2}$**	0.5863	5.6693	4750.3480	4895.3990
14	**RSF${}_{2}^{3}$**	0.4972	4.7460	3973.9930	4146.2930
15	**RSF${}_{2}^{4}$**	0.4956	4.7298	3960.3820	4133.2500
16	**RSF${}_{3}^{0}$**	0.2988	2.6629	2255.5920	2539.5080
17	**RSF${}_{3}^{1}$**	0.2962	2.6345	2231.6820	2517.1330
18	**RSF${}_{3}^{2}$**	0.2203	1.8700	1585.7510	1874.4260
19	**RSF${}_{3}^{3}$**	0.2971	2.6441	2239.7430	2524.6700
20	**RSF${}_{3}^{4}$**	0.2985	2.6594	2252.5970	2536.7020
21	**RSF${}_{4}^{0}$**	0.2392	1.9147	1593.3890	1980.4850
22	**RSF${}_{4}^{1}$**	0.2416	1.9428	1616.9220	2000.8470
23	**RSF${}_{4}^{2}$**	0.3201	2.8522	2381.5920	2659.1560
24	**RSF${}_{4}^{3}$**	0.2408	1.9332	1608.8620	1993.9700
25	**RSF${}_{4}^{4}$**	0.2395	1.9182	1596.2950	1983.0290

**Note:** Regression spline filter (RSF). In the forecasting models, we assign a code to each model: “0” for the ANN, “1” for the SVM1, “2” for the SVM2, “3” for the RF, and “4” for the DT. For example, RSF${}_{1}^{0}$ represents the estimate of the long-term trend (L_t_) with the ANN and the residual series (R_t_) estimated using the SVM1.

**Table 3:** World Monkeypox Virus Data: out-of-sample one-day-ahead mean forecast error for all combination models using the SS filter.

S.No	Models	RMSPE	MAPE	MAE	RMSE
1	**SSF${}_{0}^{0}$**	0.2453	1.9858	1653.0550	2031.8290
2	**SSF${}_{0}^{1}$**	0.2457	1.9904	1656.9690	2035.2010
3	**SSF${}_{0}^{2}$**	0.2732	2.3178	1932.1830	2265.4930
4	**SSF${}_{0}^{3}$**	0.2440	1.9712	1640.8210	2021.3110
5	**SSF${}_{0}^{4}$**	0.2449	1.9812	1649.2080	2028.5190
6	**SSF${}_{1}^{0}$**	0.1439	1.2690	1067.9890	1211.4960
7	**SSF${}_{1}^{1}$**	0.1438	1.2680	1067.0680	1210.6070
8	**SSF${}_{1}^{2}$**	0.1415	1.2162	1020.2140	1182.9890
9	**SSF${}_{1}^{3}$**	0.1442	1.2722	1070.8680	1214.3620
10	**SSF${}_{1}^{4}$**	0.1440	1.2700	1068.8940	1212.3830
11	**SSF${}_{2}^{0}$**	0.4928	4.7009	3936.0090	4109.9020
12	**SSF${}_{2}^{1}$**	0.4933	4.7058	3940.1540	4113.8720
13	**SSF${}_{2}^{2}$**	0.5248	5.0340	4216.1620	4378.9440
14	**SSF${}_{2}^{3}$**	0.4914	4.6855	3923.0550	4097.4980
15	**SSF${}_{2}^{4}$**	0.4924	4.6960	3931.9360	4106.0020
16	**SSF${}_{3}^{0}$**	0.2833	2.4950	2114.1720	2408.0140
17	**SSF${}_{3}^{1}$**	0.2828	2.4904	2110.2570	2404.4040
18	**SSF${}_{3}^{2}$**	0.2549	2.2077	1871.5680	2168.3610
19	**SSF${}_{3}^{3}$**	0.2846	2.5095	2126.4060	2419.3060
20	**SSF${}_{3}^{4}$**	0.2837	2.4996	2118.0190	2411.5630
21	**SSF${}_{4}^{0}$**	0.2392	1.9151	1593.7240	1980.7790
22	**SSF${}_{4}^{1}$**	0.2396	1.9197	1597.5240	1984.1050
23	**SSF${}_{4}^{2}$**	0.2668	2.2426	1868.9750	2211.8310
24	**SSF${}_{4}^{3}$**	0.2380	1.9010	1581.8500	1970.4030
25	**SSF${}_{4}^{4}$**	0.2388	1.9107	1589.9910	1977.5130

**Note:** Smoothing spline filter (SSF). In the forecasting models, we assign a code to each model: “0” for the ANN, “1” for the SVM1, “2” for the SVM2, “3” for the RF, and “4” for the DT. For example, SSF${}_{1}^{0}$ represents the estimate of the long-term trend ($L_{t}$) with the ANN and the residual series ($R_{t}$) estimated using the SVM1.

**Table 4:** World Monkeypox Virus Data: out-of-sample one-day-ahead mean forecast error for all combination models using the HP filter.

S.No	Models	RMSPE	MAPE	MAE	RMSE
1	**HPF${}_{0}^{0}$**	0.2476	2.0125	1675.5110	2050.9090
2	**HPF${}_{0}^{1}$**	0.2468	2.0035	1667.9020	2044.5200
3	**HPF${}_{0}^{2}$**	0.3663	3.3588	2807.5720	3046.5700
4	**HPF${}_{0}^{3}$**	0.2474	2.0105	1673.7750	2049.4510
5	**HPF${}_{0}^{4}$**	0.2478	2.0152	1677.7820	2052.8180
6	**HPF${}_{1}^{0}$**	0.1530	1.3411	1131.7320	1294.3660
7	**HPF${}_{1}^{1}$**	0.1533	1.3435	1133.9060	1297.5640
8	**HPF${}_{1}^{2}$**	0.1605	1.2661	1055.2190	1329.2900
9	**HPF${}_{1}^{3}$**	0.1531	1.3416	1132.2280	1295.0920
10	**HPF${}_{1}^{4}$**	0.1529	1.3403	1131.0830	1293.4200
11	**HPF${}_{2}^{0}$**	0.4966	4.7406	3969.4040	4141.8950
12	**HPF${}_{2}^{1}$**	0.4958	4.7313	3961.5770	4134.3950
13	**HPF${}_{2}^{2}$**	0.6269	6.0870	5101.5360	5236.8690
14	**HPF${}_{2}^{3}$**	0.4964	4.7384	3967.6180	4140.1840
15	**HPF${}_{2}^{4}$**	0.4969	4.7434	3971.7400	4144.1340
16	**HPF${}_{3}^{0}$**	0.2952	2.6241	2222.9010	2508.9300
17	**HPF${}_{3}^{1}$**	0.2960	2.6329	2230.2930	2515.8350
18	**HPF${}_{3}^{2}$**	0.1884	1.6163	1369.1300	1602.0430
19	**HPF${}_{3}^{3}$**	0.2954	2.6261	2224.5880	2510.5050
20	**HPF${}_{3}^{4}$**	0.2950	2.6215	2220.6950	2506.8700
21	**HPF${}_{4}^{0}$**	0.2423	1.9511	1623.9360	2006.8410
22	**HPF${}_{4}^{1}$**	0.2416	1.9423	1616.5440	2000.5240
23	**HPF${}_{4}^{2}$**	0.3603	3.2943	2753.3300	2996.6570
24	**HPF${}_{4}^{3}$**	0.2421	1.9491	1622.2500	2005.3990
25	**HPF${}_{4}^{4}$**	0.2425	1.9537	1626.1420	2008.7290

**Note:** Hodrick–Prescott Filter (RSF). In the forecasting models, we assign a code to each model: “0” for the ANN, “1” for the SVM1, “2” for the SVM2, “3” for the RF, and “4” for the DT. For example, HPF${}_{1}^{0}$ represents the estimate of the long-term trend ($L_{t}$) with the ANN and the residual series ($R_{t}$) estimated using the SVM1.

**Table 5:** World Monkeypox Virus Data: the best final combination models results of the out-of-sample cumulative confirmed day-ahead mean forecast error.

S.No	Models	RMSPE	MAPE	MAE	RMSE
1	**RSF${}_{1}^{2}$**	0.1452	1.2083	1010.7360	1207.9950
2	**SSF${}_{1}^{2}$**	0.1455	1.2162	1020.2140	1222.9890
3	**HPF${}_{1}^{2}$**	0.1605	1.2661	1055.2190	1329.2900

**Table 6:** World Monkeypox Virus Data: results (*p*-value) of the DM test for the null hypothesis that the two models on the rows and columns are equally accurate and the alternative hypothesis that the model on the columns is more accurate than the model on the rows (using the loss square function).

Models	RSF${}_{1}^{2}$	RSF${}_{1}^{2}$	RSF${}_{1}^{2}$
**RSF${}_{1}^{2}$**	-	0.18	1.00
**SSF${}_{1}^{2}$**	0.82	-	0.99
**HPF${}_{1}^{2}$**	0.00	0.01	-

**Table 7:** World Monkeypox Virus Data: comparison of the proposed best model versus the considered time series and machine learning benchmark models: out-of-sample cumulative confirmed day-ahead mean forecast error.

S.No	Models	RMSPE	MAPE	MAE	RMSE
1	**RSF${}_{1}^{2}$**	0.1452	1.2083	1010.7360	1207.9950
2	**ANN**	0.2470	2.0055	1669.6410	2045.9800
3	**SVM**	0.4868	4.6378	3882.9750	4059.1410
4	**RF**	0.2826	2.4879	2108.1850	2402.4950
5	**DT**	0.2400	1.9241	1601.2560	1987.3760
6	**AR**	0.2556	2.1090	1756.6180	2117.7440
7	**NPAR**	0.2482	2.0205	1682.1830	2056.4220
8	**ARIMA**	0.2502	2.0441	1702.1040	2072.7490

**Table 8:** World Monkeypox Virus Data: the best final model and the considered time series and machine learning benchmark models: results (*p*-value) of the DM test for the null hypothesis that the two models on the rows and columns are equally accurate and the alternative hypothesis that the model on the columns is more accurate than the model on the rows (using the loss square function).

Models	RSF${}_{1}^{2}$	ANN	SVM	RF	DT	AR	NPAR	ARMA
**RSF${}_{1}^{2}$**	-	1.00	1.00	1.00	1.00	1.00	1.00	1.00
**ANN**	0.00	-	1.00	0.93	0.00	1.00	1.00	1.00
**SVM**	0.00	0.00	-	0.00	0.00	0.00	0.00	0.00
**RF**	0.00	0.07	1.00	-	0.04	0.12	0.07	0.09
**DT**	0.00	1.00	1.00	0.96	-	1.00	1.00	1.00
**AR**	0.00	0.00	1.00	0.88	0.00	-	0.00	0.00
**NPAR**	0.00	0.00	1.00	0.93	0.00	1.00	-	1.00
**ARMA**	0.00	0.00	1.00	0.91	0.00	1.00	0.00	-

**Table 9:** World Monkeypox Virus Data: the forecasted cumulative and daily confirmed cases of the monkeypox virus using the best-proposed model over two weeks.

S.No	Date	CC	DC	PFE
1	11 February 2023	84,063	77	2.129
2	12 February 2023	84,140	77	2.051
3	13 February 2023	84,189	49	1.994
4	14 February 2023	84,225	36	1.976
5	15 February 2023	84,264	39	1.956
6	16 February 2023	84,309	45	1.908
7	17 February 2023	84,356	47	1.857
8	18 February 2023	84,402	46	1.920
9	19 February 2023	84,448	46	1.866
11	20 February 2023	84,492	44	1.815
12	21 February 2023	84,537	45	1.769
13	22 February 2023	84,583	46	1.748
14	23 February 2023	84,628	45	1.777

Note: cumulative confirmed cases (CC), daily confirmed cases (DC), and percentage forecast error (PFE).

## Data Availability

Data will be provided upon the request of the first author.
